# Round the Hospitals

**Published:** 1918-01-12

**Authors:** 


### ROUND THE HOSPITALS.

The list of promotions in and appointments to the Most Excellent Order of the British Empire, dated January 1, which the King as Sovereign of the Order has been pleased to command, occupied upwards of sixteen columns of the *Times* of the 8th instant. It is manifest that we cannot attempt to give the names in full. The whole Empire, and trained nurses everywhere, will rejoice to learn that the title and dignity of a Dame Grand Cross of the Order of the British Empire has been conferred upon Her Majesty Queen Alexandra. From the outbreak of the war Her Majesty has continu-

☛ Previous articles appeared December 8, 15, 22 and January 5.

**ROUND THE HOSPITALS—(*continued*).**

ously devoted her thoughts, energies, personal service and substance to the promotion, in all available fields, of everything which has tended to help forward and perfect the provision of adequate accommodation for our wounded warriors, and to secure and extend the supply of trained nurses, to whose devoted care our sailors and soldiers never tire of bearing grateful testimony. When Princess of Wales, Queen Alexandra, in conjunction with our late lamented King Edward VII., rendered the most material service to the nursing profession, as President of the Royal National Pension Fund for Nurses, which is to-day the greatest thrift agency amongst women workers which the world contains. At the time of the South African War Her Majesty selected and sent out a number of trained nurses who rendered notable service, many of whom have been actively working in military, Territorial, and other hospitals during the present war, and have won distinguished honours by their zealous efficiency. January 1, 1918, cannot fail to be a memorable date in the life of the fortunate possessors of the dignity of a Dame Grand Cross of the Most Excellent Order of the British Empire, for on that day Her Majesty Queen Alexandra joined their ranks by command of His Majesty the King. Queen Alexandra has one of the tenderest and kindest hearts that ever beat in the breast of woman. Her life amongst her beloved people, within the British Empire, has won for her a personal affection and devotion so deep and spontaneous, that, it must constitute the most precious possession which a monarch can hope to win in this world. Her kindly and spontaneous visit at Christmas to the children who were victims of the air-raids, which we have already recorded, is but the latest example of Queen Alexandra's continuous sympathy with the suffering, to whom that sympathy is precious beyond words.

The British Women's Hospital Committee, with great self-denial, are rendering such splendid service to the nursing profession by raising money for the College buildings, its scholarships, and educational work for nurses, in addition to a Tribute Fund as a thankoffering from the British Empire to British nurses, that everything which confers honour and recognition upon such services must be welcome news for trained nurses. The whole nursing profession will rejoice to hear that His Majesty the King, as Sovereign of the Order, has given orders for the appointment of Mrs. May Webster, Chairman of the British Women's Hospital Committee, to be a Dame Commander (D.B.E.) of the Most Excellent Order of the British Empire, for services in connection with the war. Few people are in a position to know, the volume of continuous personal service and genuine hard work entailed upon the British Women's Hospital Committee and its officers, in connection with their self-denying and spontaneous efforts to place the nursing profession upon a foundation of financial strength, equivalent to that provided for the profession of medicine, by eleemosynary gifts. These essential endowments, and other progressive developments for the Nursing Profession, might otherwise have been absent. Had there been any doubt of the wisdom of the step taken by the British Women's Hospital Committee and their public service in raising a thankoffering from the British Empire to British nurses, the unjustifiable and outrageous conduct of a few women, with personal axes to grind, would have dissipated such doubts, as, indeed, they have done, by exhibiting the unsubstantiality and absurdity of this hostile action to all classes throughout the country. All honour, then, to Mrs. May Webster, Lady Cowdray, and their colleagues, who will rejoice with the nurses, that, their Chairman has been included by her Sovereign in the list of New Year Honours. We may add that our cousins and Allies, being hospital workers in the United States of America, will doubtless share the satisfaction caused by His Majesty's action, and heartily join in the Empire's congratulations to Dame May Webster and the ladies who constitute the British Women's Hospital Committee.

In the Supplement to the *London Gazette* of January 1 the announcement appeared that the King has been pleased to award a bar to the Royal Red Cross to the following ladies of the Nursing Service in recognition of their very valuable services during the war:—Miss Ethel Hope Becher, R.R.C., Matron-in-Chief, Q.A.I.M.N.S.; Miss Sidney Jane Browne, R.R.C., Matron-in-Chief, T.F.N.S. (ret., Q.A.I.M.N.S.); Miss Jane Hoadley, R.R.C., Matron, Q.A.I.M.N.S.; Miss Beatrice Isabel Jones, R.R.C., Matron, Q.A.I.M.N.S.; Miss Emma Maud McCarthy, R.R.C., Matron-in-Chief, Q.A.I.M.N.S.; Miss Sarah Elizabeth Oram, R.R.C., Principal Matron (T. Matron-in-Chief), Q.A.I.M.N.S.; Miss Annie Beadsmore Smith, R.R.C., Principal Matron, Q.A.I.M.N.S.; Miss Mary Wilson, R.R.C., Principal Matron, Q.A.I.M.N.S.

In The Hospital of July 7, 1917, p. 284, we published an address from the Nightingale Sisters and hundreds of nurses connected with St. Thomas's Hospital to Mr. J. G. Wainwright, the retiring treasurer, who had been for twenty-seven years the administrative head of the nurses and the Nightingale School. At Christmas Mr. Wainwright received a further letter signed by those matrons, assistant matrons and sisters who had been fellow-workers with himself at St. Thomas's Hospital during his long service there as treasurer. The signatories include a large number of the most active and best-known matrons and members of the nursing profession, who have had the privilege of being trained at the Nightingale School. Naturally this Christmas packet gave Mr. Wainwright infinite pleasure, and in his reply he writes: "To have gained the affectionate feelings which this letter conveys, is to me a most precious memento of a most happy life, surrounded as it has been by the hearty and unfailing co-operation of all who have held responsible posts in our (St. Thomas's) Hospital." Nothing can be more admirable than the spirit of unity and pride in their

**ROUND THE HOSPITALS—(continued).**

Alma Mater, which has ever characterised the army of noble women who obtained their entrance to the nursing profession through the Nightingale School. Mr. Wainwright is deservedly honoured and beloved by his nurses, and all hospital workers will cordially enter into the feelings of happiness which he experienced this Christmastide, on the receipt of the memorable expression of good-will which it is our privilege to report.

THE press of all shades of opinion have extended generous support to the British Women's Hospital Committee's movement to raise money for the College buildings, its scholarships and educational work for nurses, and the Tribute Fund. As a consequence, in accordance with the invariable practice of personalities and abuse pursued by the originating centre of organised attempts to create disunion, within the nursing profession, it has, with tactless absurdity, denounced the newspapers, including some of the most influential of their number, as "a subsidised press." This ludicrous policy owes its origin to chagrin, caused by the refusal to give publicity to certain letters, written from this centre of unworthiness, by some of the small band of malcontents, who during 1917 revealed themselves in the columns of the *Sunday Times*, which rendered public service by thus exposing the nature of this intrigue, and the insignificant numbers of the active participants in it. The absurdity of charging the press, with a "press boycott" in nursing affairs, has been disproved in several directions lately. Thus the *Yorkshire Post* opened its columns recently to a discussion originating with members of the band, and enabled correspondents who carry great weight in the nursing profession to reply to them with telling force. By this publicity residents throughout the North of England have been enabled to realise the instability and misrepresentation, on which the fulminations of these would-be disturbers of the peace of the nursing profession, rest. By this action the *Yorkshire Post* has rendered good service to the nation, and won the gratitude of trained nurses also.

Two members of the Council of the College of Nursing, Misses M. E. Sparshott, R.R.C., and C. E. Vincent, R.R.C., published in the *Yorkshire Post* of the 26th ulto. some telling home truths dealing with the band's insistence on what it calls "Nurses' self-governing organisations." They ask, "Have these societies ever published any list of their membership, even though the subscription of the Society for the State Registration of Nurses is put at the attractive sum of 1s. per annum? Is there anything to prevent a nurse joining the whole group of these societies, and thus being counted as many times over as there are societies?" We may here point out that Miss Jentie B. Paterson, a supporter of the band's views, states, that, when invited to become a member of the Scottish Nurses' Association she refused, on the grounds that, as she was a member of the N.U.T.N. and the Society for the State Registration of Trained Nurses, and as "we were all fighting for the same cause and represented on the Central Committee," she was already *ipso facto* a member. This suggests an active attempt to induce a nurse to join the whole group of these societies, which, if generally successful, might make the total membership appear to be many times greater than it is in fact, by counting each separate member as many times over as there are so-called "self-governing organisations."

ANOTHER point which the band make the most of is that a Bill for the State Registration of Nurses was once passed by the House of Lords. They fail to mention that the Bill promoted by the Central Committee reached a first reading in the Commons, only after a division, and it is well known that unless a Bill is distinctly contentious the first reading is practically never challenged. "The actual progress" (and this is really rather pathetic," Misses Sparshott and Vincent add) "made after twenty years of effort by Mrs. Bedford Fenwick and her friends, may be summed up as a Bill for the State Registration of Nurses, which in the House of Commons was not even permitted to go to a first reading without challenge. All honour to them for what they have done and attempted to do, but why should they regard with jealousy and hostility the efforts of others to bring other weapons to bear, and to launch the attack from another quarter? for be it remembered that the College of Nursing is pledged to promote a Bill for State Registration, no less deeply, than the nurses' societies championed by Miss Eden."

MISSES SPARSHOTT AND VINCENT proceed: "Mrs. Bedford Fenwick began her campaign years ago with enlisting the help of the nurses working for themselves, and scattered throughout the country. For this she has deserved well of the nursing profession, though few will approve her methods. What Sir Arthur Stanley has done is to start from the nurse-training schools, not only those connected with the voluntary hospitals, but those which have to do with the Poor-Law infirmaries, basing himself, in fact, upon the educational bodies just as the General Medical Council, of which Miss Eden speaks, is a representative and independent body, based upon the Universities and Colleges which train medical students for doctors, but with this difference, that whereas the medical profession at large appoints only six members on the General Medical Council out of about thirty, the Council of the College of Nursing is entirely elective, and by next April one-third will have been elected by the nurses who are members of the College. How could Sir Arthur Stanley and those with him have shown greater confidence in the nurses, than by placing the College, at the very earliest moment that its membership had become substantial and representative, upon a frankly democratic basis and with a due proportion of the Council chosen for Scotland and Ireland?"

ONCE again they proceed: "Why does Miss Eden describe the honorary officers of the College of Nursing as 'three men who are definitely

**ROUND THE HOSPITALS—(*continued*).**

employers'? Only apparently to import prejudice by implying that they have interests contrary to the interests of nurses. Sir Arthur Stanley, the chairman of the Council, is also chairman of the Committee of the Red Cross Society, and only since the College was formed has he become the treasurer of St. Thomas's Hospital. Why should he be pilloried as an employer? What interests has he opposed to those of the nurses? And what does the College of Nursing do for him except to take an enormous amount of his time, thought, and labour? Sir Cooper Perry, the honorary secretary, is himself 'employed' at Guy's Hospital. Why should he be unable to sympathise with the needs of those other employed people, amongst whom he has lived and worked for the last thirty years? Mr. Comyns Berkeley, the honorary treasurer, has been treasurer for many years past of the Royal British Nurses' Association. Would he have been ever so appointed, or continued in his appointments, if he had not been a champion of nurses, who elected him to that honourable position?"

Miss Sparshott and Miss Vincent further proceed to make certain truths plain. "It is not the fact that the College has hitherto refused 'any representation whatever' to the nurses' societies, in its Bill for State Registration. Sir Arthur Stanley originally offered the Central Committee the same number of members on the General Nursing Council to be set up under the Bill, as the College was to have. He asked the Central Committee to let him have the names of the nurses whom they desired to nominate, so as to set them out in the draft Bill. The Central Committee at this point themselves broke off negotiations, on the ground that they must insist upon having the names of the societies, and not the names of the persons to represent the societies, set out in the Bill. Sir Arthur replied that as a matter of policy and tactics he thought they were wrong, but in his view no vital principle was concerned, nor any difference that could not be easily solved by men and women of good will." "Meanwhile," add Miss Sparshott and Miss Vincent, as the columns of the *Yorkshire Post* have shown, "the College of Nursing comes in for all this hostile action on the part of those who, no less than the College, are aiming at the State Registration of Nurses. Is this the way to get it?" In conclusion they justifiably claim that they have stated "enough to show how ill-informed and prejudiced is the criticism in the correspondence columns of the *Yorkshire Post*, and elsewhere." We are confident that every impartial member of the nursing profession, not only in this country, but throughout the Empire and in the United States of America, will feel grateful to Miss M. E. Sparshott and Miss C. E. Vincent for the terse and able way in which they have revealed the true inwardness of the methods of those, who are wholly responsible for the existing attempt to promote disunion amongst trained nurses. They are, in fact, the real impediment to the immediate granting of State Registration to trained nurses throughout the United Kingdom.

The Council of the College is already making preliminary arrangements for the Council election, which will be held next April, when one-third of the present members of the Council retire. Within a short time, nomination papers must be sent out to the members to afford ample time for their return, and the preparation of the voting papers. Members who have paid the fees are eligible to vote, and will receive a nomination paper in due course. In reply to inquiries, we may add that the latest date by which it will be necessary for nurses to have paid their fees to qualify them to receive a nomination paper, will be announced shortly. Prompt payment is therefore good business in this as in all things. Members who have failed to notify the Secretary of any change in their present and (or) permanent home address, are asked to send particulars to 6 Vere Street, Cavendish Square, W. 1, immediately.

☛ It is our invariable practice to pay for all items of news which we may publish. The minimum payment is 5s. Paragraphs of about twenty lines in length—that is to say, of 150 words or less—are preferred. Contributions should be addressed to The Editor, The Hospital, 28 and 29 Southampton Street, Strand, London, W.C. 2, and, to be in time for the current week's issue, should reach him by the first post on Mondays.

For Nursing Affairs in Ireland, see p. 316.

